# Predictors for outcome in acute lateral epicondylitis

**DOI:** 10.1186/s12891-019-2758-y

**Published:** 2019-08-17

**Authors:** Øystein Holmedal, Morten Olaussen, Ibrahimu Mdala, Bård Natvig, Morten Lindbæk

**Affiliations:** 1 Researcher, Department of General Practice, Institute of Health and Society, University of Oslo, Oslo, Norway; 2 Department of General Medicine, University of Oslo, PO Box 1130, Blindern, NO-0318 Oslo, Norway

**Keywords:** Lateral epicondylitis, Treatment, Predictors, General practice

## Abstract

**Background:**

Lateral epicondylitis or tennis elbow is a frequent condition with long-lasting symptoms. In order to identify predictors for treatment success and pain in lateral epicondylitis, we used data from a randomized controlled trial. This trial investigated the efficacy of physiotherapy alone or combined with corticosteroid injection for acute lateral epicondylitis in general practice.

**Methods:**

The outcomes treatment success and pain score on VAS were assessed at 6, 12, 26 and 52 weeks. We ran a univariate binary logistic regression with generalized estimating equations (GEE) and subsequently an adjusted multilevel logistic regression to analyze the association between potential prognostic indicators and the outcome success/ no success. To assess the changes in pain score we used a two-level multilevel linear regression (MLR) followed by an adjusted MLR model with random effects.

**Results:**

The most consistent predictor for reduced treatment success at all time points was a high Pain Free Function Index score signifying more pain on everyday activities. Being on paid sick-leave and having a recurring complaint increased short term treatment success but gave decreased long-term treatment success. The patients reporting symptoms after engaging in probable overuse in an unusual activity, tended towards increased treatment success at all time-points, but significant only at 12 weeks. The most consistent predictor of increased pain at all time points was a higher overall complaints score at baseline.

Conclusions: Our results suggest that in treating acute lateral epicondylitis, a consideration of baseline pain, a registration of the patient’s overall complaint on a VAS scale and an assessment of the patient’s perceived performance in everyday activities with the Pain Free Function Index can be useful in identifying patients that will have a more protracted and serious condition.

**Trial registration:**

ClinicalTrials.gov Identifier: NCT00826462. Date of registration January 22, 2009. The Trial was prospectively registrated.

**Electronic supplementary material:**

The online version of this article (10.1186/s12891-019-2758-y) contains supplementary material, which is available to authorized users.

## Background

Lateral epicondylitis of the elbow is frequently encountered in general practice with an incidence of 5.5-person years [1]. It is characterized by pain and tenderness over the lateral humeral epicondyle and pain on resisted dorsiflexion and radial deviation of the wrist. The complaint often resolves spontaneously in 6–12 months [2], but many patients suffer considerable pain and discomfort and need time off from work. One of the latest published papers on predictors for outcome in lateral epicondylitis from 2006 [3], based on trial results published in 1999 and 2002, found that long duration of elbow complaints, concomitant neck pain and severe pain at presentation are associated with poorer outcome at 12 months. This is consistent with what other investigators have found [4–8]. Patients from higher social classes reported lower pain scores at 1 year than patients from lower social classes. In addition, other authors have found that factors predicting worse outcome for pain at 12 months are female gender [6, 9], higher age [7, 8, 10], recurrent complaint [4, 5] dominant arm affected [5, 7], and manual work [7–9].

### Objective

The objective of this paper was to identify predictors for treatment success and pain relief in acute lateral epicondylitis at 6, 12, 26 and 52 weeks follow-up using data from a recently published treatment study with stringent inclusion criteriae, a family practice setting and a 1-year follow-up [11].

## Materials and methods

### Study design

In this paper, we used data from a randomized, controlled study published in 2015 [11], where we investigated the effect of physiotherapy with or without corticosteroid injections against a control group with no treatment on acute lateral epicondylitis. We followed 177 patients aged 18 to 70 with recent onset lateral epicondylitis for 1 year. The main outcome measure was treatment success defined as patients rating themselves completely recovered or much better on a six-point scale. A number of secondary outcomes were registered, including pain on a Visual Analogue Scale (VAS). We registered all these variables as patient characteristics at baseline and as outcomes at six, 12, 26 and 52 weeks. The methods for registering outcomes are described in detail in our original paper (treatment success, pain, affected function, overall complaint, pain-free and maximum grip, pain on resisted dorsiflexion of the wrist and third finger, pain on eight every-day activities). In the investigation of prognostic indicators, we chose to use the primary outcome of treatment success, and as secondary outcome elbow pain measured on VAS. The variables registered throughout the study were used in statistical analyses to identify which of these had any significant influence on treatment success and pain.

### Statistical method

Treatment success was registered at four follow-up time points (6, 12, 26 and 52 weeks). These repeated measurements were assumed to be correlated within a patient. Therefore, analysis methods that assume independence of observations were rendered inappropriate. We modelled the binary response of success/ no success using the binary logistic regression with generalized estimating equations (GEE) to handle the dependence in the data. The exchangeable correlation structure was considered for the GEE binary logistic models. First, we assessed the effects of each patient-level prognostic factor on success over time. Secondly, all prognostic factors in step 1 with *P* ≤ 0.20 and *P* ≤ 0.05 at any of the time points 6 weeks, 12 weeks, 26 weeks and 52 weeks in addition to baseline scores were used to fit two separate final models. Model selection was based on the independence model criterion (QIC), which seeks the model with the smallest estimate. Repeated measurements of pain score were obtained at baseline and at the same four follow-up time points and were assumed to be correlated within a patient. We considered a two-level multilevel linear regression (MLR) model with random effects of time (occasions) as level-1 units and patients at level-2 to assess the changes in pain score. We used the same steps as described above to model pain score. In addition, an estimate of intracluster correlation (ICC) was obtained from the adjusted final fit. The ICC helped to explain the amount of variability in pain score attributable to differences between the patients. At each stage of the model development, the Akaike Information Criteria (AIC) was used to check if the inclusion of a variable or variables improved the model fit. The AIC states that given a set of candidate models for the data, the model with the smallest AIC value should be considered a better fit. Therefore, we used the AIC to select a better fit of pain score. For both outcomes, age and gender were included in the final models as clinically relevant regardless of their significance level. All the models were fitted using StataSE 14 and the significance level of the final models were set at *p* = 0.05.

## Results

The baseline characteristics of the patients are presented in Table 1.

**Table 1:** Baseline characteristics of the study participants

Socio-demographic characteristics		Total
		177
Age	*Years, mean ± SD*	47.0 ± 9.7
Women		71 (40.1)
Marital status		
*Unmarried/ widow (er)*		44 (24.9)
*Married/ cohabiting*		133 (75.1)
Education		
*No higher education (primary/ secondary school)*		124 (70.1)
*Higher education (college/ university)*		52 (29.4)
Excercises regularly		86 (48.6)
Paid work		154 (87.0)
Manual labor		100 (56.5)
On paid sick-leave now?		51 (28.8)
Duration of complaints	*Weeks, mean ± SD*	7.0 ± 3.1
Dominant elbow affected?		126 (71.2)
Pain every day last week?		170 (96.0)
Use of analgesics last week		55 (31.1)
Acute start?		94 (53.1)
Similar complaints earlier		41 (23.2)
Probable over-use usual activity?		110 (62.1)
Probable over-use unusual activity?		65 (36.7)
Patients preference for treatment: Physiotherapy		67 (37.9)
Patients preference for treatment: Wait and see		11 (6.2)
Patients preference for treatment: Injection		40 (22.6)
Patients preference for treatment: No preference		58 (32.8)
Pain score on VAS (0–100 mm)	*Mean ± SD*	52.2 ± 20.4
Affected function on VAS	*Mean ± SD*	53.4 ± 21.9
Overall complaints on VAS	*Mean ± SD*	64.1 ± 19.2
Pain Free Grip Strength Ratio (affected/ unaffected arm)	*Mean ± SD*	0.36 ± 0.27
Max Grip Strength Ratio (affected/ unaffected arm)	*Mean ± SD*	0.73 ± 0.34
Pain Free Function Index	*Mean ± SD*	5.8 ± 2.0
Pain-free isometric dorsiflexion of wrist		
*None*		2 (1.1)
*Some or distinct*		175 (98.9)
Pain-free isometric extension of third finger		
*None*		14 (7.9)
*Some or distinct*		162 (91.5)

**Fig. 1:**
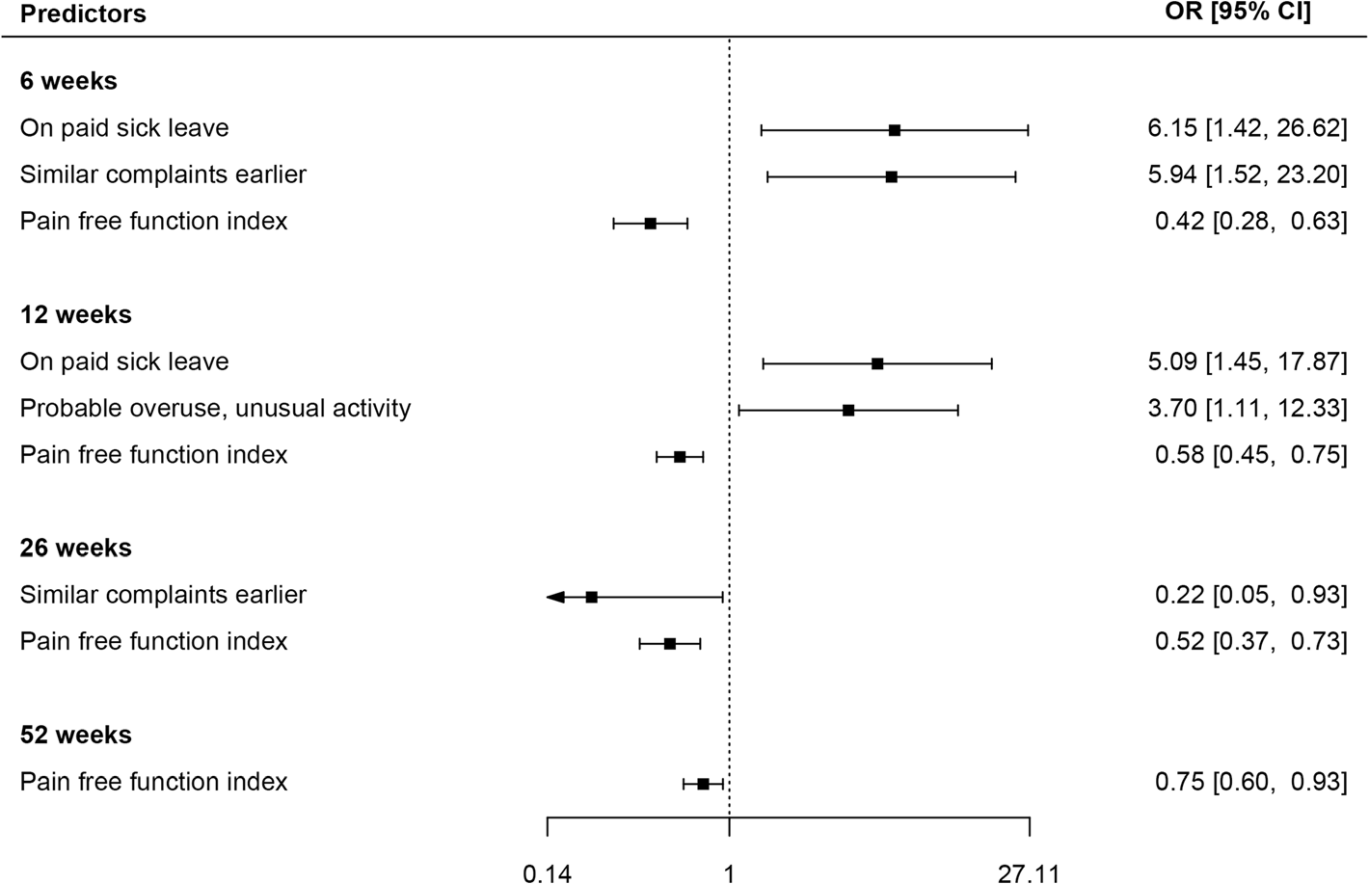
Forest plot of the statistically significant predictors for treatment success. Odds ratio with 95% confidence interval

**Fig. 2:**
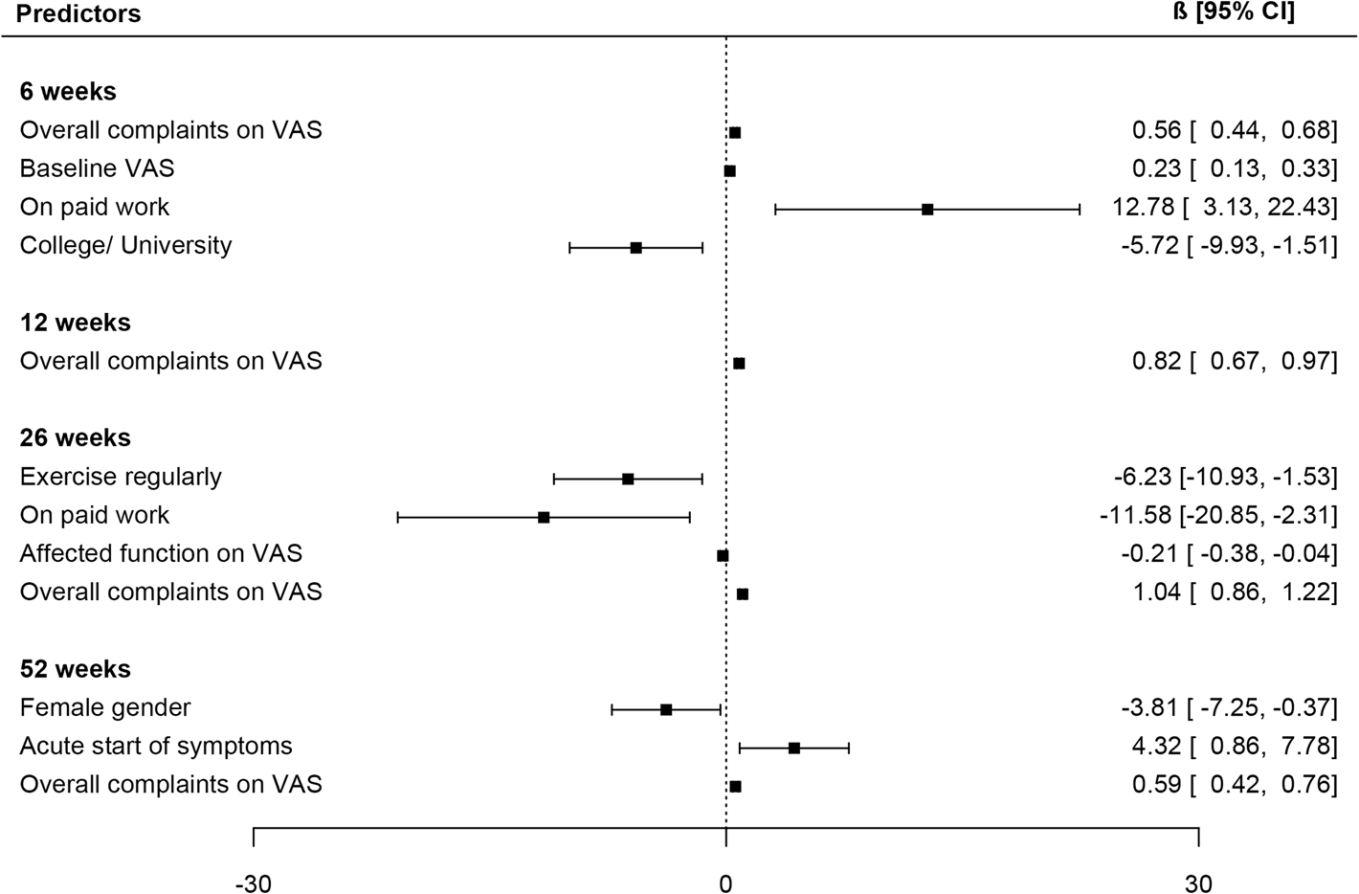
Forest plot of the statistically significant predictors for changes in pain. Mean change of pain on VAS with 95% confidence interval

The mean age at baseline was 47 years, 40% were women, and the mean duration of complaints was 7 weeks. The registered pain score on VAS was 52, the Pain Free Function score was 5.8 and the variable Overall complaints on VAS was 64. The effect of prognostic variables on treatment success is presented in Additional file 1: Table S2 and Additional file 2: Table S3. Additional file 1: Table S2 shows the unadjusted effect of each prognostic factor on treatment success obtained from the GEE binary logistic regression Additional file 2: Table S3 shows adjusted estimates of odds ratios (OR) and their 95% confidence intervals obtained from the analysis of prognostic indicators of success using the GEE binary logistic regression model. We obtained an ICC estimate of 0.061 from the full model of pain. An ICC = 0.061 means that differences between patients accounted for 0.061 × 100% = 6.1% of the variability in pain score. The effect of prognostic variables on pain is presented in Additional file 3: Table S4 and Additional file 4: Table S5. Additional file 3: Table S4 shows the univariate multilevel linear regression analysis of prognostic indicators on pain. Additional file 4: Table S5 shows the association between prognostic indicators and pain at each study time point obtained from an adjusted multilevel linear regression (MLR) model. A summary of the statistically significant findings at each follow-up is given as forest plots for treatment success in Fig. 1 and for pain in Fig. 2.

### Prognostic indicators at 6 weeks

Patients on paid sick leave at baseline and those having had epicondylitis earlier at baseline were more likely to have treatment success at 6 weeks follow-up (Additional file 2: Table S3). Patients with a higher Pain Free Function Index score were less likely to have treatment success (higher Pain Free Function Index means pain on more activities). Pain score was significantly lower among participants with higher education than those with lower education (Additional file 4: Table S5). A higher pain score was observed among participants with paid work compared to those without paid work and for those with a higher level of baseline pain and overall complaint.

### Prognostic indicators at 12 weeks

Patients on paid sick leave at baseline and those reporting probable overuse of their arm in an unusual activity were more likely to report treatment success at 12 weeks (Additional file 2: Table S3). Patients with a higher Pain Free Function Index score were less likely to have treatment success. A higher overall complaint score at baseline gave a significantly higher pain score at 12 weeks (Additional file 4: Table S5).

### Prognostic indicators at 26 weeks

Patients with epicondylitis earlier and those with an increased Pain Free Function Index score were less likely to register treatment success at 26 weeks follow-up (Additional file 2: Table S3). Females were more likely to report treatment success. We observed a significantly lower pain score among participants who exercised regularly as well as participants on paid work. It was further observed that a higher affected function score at baseline was associated with a lower pain score at 26 weeks. A higher overall complaints score was associated with an increase in pain score.

### Prognostic indicators at 52 weeks

Patients with a higher Pain Free Function Index score (pain at more activities) were less likely to have treatment success at 52 weeks (Additional file 2: Table S3). Females had a lower pain score compared to males (Additional file 4: Table S5). An acute start of symptoms and higher overall complaints were significantly associated with a higher pain score after 52 weeks.

## Discussion

We found that a high Pain Free Function Index score at baseline was a predictor for a lower rate of treatment success at all time points. The most consistent predictor for more pain at all time points was baseline overall complaints.

### Treatment success

The most consistent predictor for a lower rate of treatment success at all time points was a high Pain Free Function Index score at baseline. A high score on this index of pain at 8 every day activities at baseline indicates a more serious condition, and we found that this reduces success at all time points. This is consistent with the findings of Haahr [7]. Bot [4] found a worse outcome for functional disability at 12 weeks for those with more intense pain at baseline, and at 52 weeks for those with less pain at baseline. However, Bot found that those more disabled at baseline had a better outcome at 12 weeks, whereas we found that a higher Pain Free Function index at 12 weeks reduced success. Being on paid sick-leave at baseline was significantly associated with higher rate of treatment success at 6 and 12 weeks. A possible explanation might be that resting the elbow is beneficial. Another possibility is that these patients were in more pain and were more likely to need sick-leave. People with a similar complaint earlier had a higher rate of treatment success at 6 weeks, but a lower success rate at 26 weeks. This might indicate that the patient recognized the problem earlier and quickly reduced exposure to harmful activities. Having a recurring problem might also suggest that the patient had a more chronic or severe condition [4, 5]. The patients relating their tennis elbow to unusual use had a higher rate of treatment success at 12 weeks. One might speculate that the patient refrained from an activity thought to be harmful. Apart from a higher success rate in females at 26 weeks, we found no association between treatment success and age, gender or paid or manual work. This is consistent with the findings of Smidt [3] regarding manual work, and Gerberich regarding gender [6].

### Pain

A high baseline overall complaints score predicted more pain at all time points. This reflects the results from other studies [3, 7]. We found no association between age or duration of complaint and pain, nor whether dominant arm was involved. Haahr [7] found that involvement of dominant elbow and age over 40 years predicted a continued high pain score. These variables did not reach significance at the univariate level in our study. Paid work and exercising regularly was associated with a lower pain score at 26 weeks, suggesting a beneficial effect of staying active. Also, we found no relation to manual work where other authors found a correlation Lewis [9] found higher pain scores at 6 months and higher function scores at 4 weeks and 6 months. Female gender predicted less pain at 52 weeks. This gender-difference is difficult to explain. Haahr [7] found no relation between gender and general improvement. This is consistent with our findings. Higher education predicted less pain at 6 weeks. This is consistent with what others have found and may indicate a correlation with higher socio-economic class [3], whereas Haahr [7] found that higher education was not related to general improvement. One might speculate that any positive effect is due to less heavy or strenuous work for people with higher education or better coping abilities. Exercising regularly gave less pain at 26 weeks. This might be due to beneficial effects of exercise on lateral epicondylitis in the long term. Acute start of symptoms meant more pain at 52 weeks. One might think that an acute start more often is caused by a sudden or heavy load or unusual use of the elbow and thus a more serious complaint. A high baseline pain score indicated more pain at 6 weeks. The more pain at baseline, the more serious and thus longer lasting complaint one might expect. This is consistent with what others have found [3–8]. A higher affected function score at baseline indicated less pain at 26 weeks. Overall complaints score indicated significantly more pain at all time points, again signifying a more serious complaint. We found no correlation between pain and baseline Pain Fee Function Index, which had a strong correlation with rate of treatment success. It is interesting that for success, the registrations of pain, affected function and overall complaint on VAS did not reach a level of significance. One might speculate that treatment success is a more qualitative outcome, corresponding with how well the participants do on everyday activities as registered with the Pain Free Function Index, whereas VAS registrations might be a more quantitative, clinical assessment leaving less latitude for a feeling of improvement.

### Strengths and limitations

This paper is based on data from a large randomized controlled trial [11], which had a general practice setting making the results correspondingly relevant. As possible predictors we selected measurements in use at the time of our original investigation. Since then, advances in musculoskeletal ultrasound have shown promising results that could lead to additional predictors of symptom duration, staging and outcome in lateral epicondylitis. (Superb Microvascular Imaging, SMI). [12].

We used a stringent selection and definition of study population complying with the Delphi List [13], a length of follow-up with multiple assessments of symptoms and severity reflecting the natural trajectory of the complaint [2, 14]. As recovery of soft tissue injuries is faster in the early stages of disease, we only included acute onset lateral epicondylitis, ensuring a homogeneous sample. Our original study was not designed to investigate predictors. This limited the choice of statistical analyses in this paper, since small subgroups would create statistically weak or invalid results. We did not investigate neck pain, or other musculoskeletal complaints, nor did we run a separate analysis as to the type of paid work (strenuous, repetitive, etc.) in relation to our chosen outcomes. Emotional, psychosocial factors or coping skills were also not investigated. We investigated recent onset lateral epicondylitis. This limited our assessment of the impact of duration of the condition on outcome.

## Conclusions and implications

The one consistent predictor of reduced success at all time points, was a high Pain Free Function Index score. The most consistent predictor for reduced pain at all time points was a low baseline score on overall complaints on VAS. Our results suggest that in treating acute lateral epicondylitis, a consideration of overall complaints and an assessment of the patient’s perceived performance in everyday activities with the Pain Free Function Index are useful in identifying patients early in the clinical course where a more protracted and serious course might be expected.

## Additional files

Additional file 1: **Table S2.** Univariate logistic showing the effects of each prognostic indicator on treatment success. (PDF 30 kb)

Additional file 2: **Table S3.** Adjusted multilevel logistic regression showing the effects of each prognostic indicator on treatment success (based on *P* ≤ 0.05 from the univariate analysis). (PDF 96 kb)

Additional file 3: **Table S4.** Univariate multilevel linear regression (MLR) showing the effects of each prognostic indicator on Pain Score (VAS) at each study time point after adjusting for baseline pain on VAS. (PDF 114 kb)

Additional file 4: **Table S5.** Adjusted MLR showing the effects of each prognostic indicator on pain (VAS) at each study time point (based on *P* ≤ 0.20 from the univariate analysis). (PDF 192 kb)

## Data Availability

Data and materials can be provided by contacting the author.

## Abbreviations

AIC: Akaike Information Criteria
GEE: Generalized estimating eqs.
ICC: Intracluster correlation
MLR: Multilevel linear regression
PFF: Pain free function index
QIC: Independence model criterion
VAS: Visual analog scale
