# Supplementary material for: Predictors for outcome in acute lateral epicondylitis
Source: BMC Musculoskelet Disord. 2019 Aug 17;20:375. doi: 10.1186/s12891-019-2758-y (PMC6698329; doi:10.1186/s12891-019-2758-y)
Supplement: Supplementary file 1 — Table S2. Univariate logistic showing the effects of each prognostic indicator on treatment success. (PDF 30 kb) [file 12891_2019_2758_MOESM1_ESM.pdf]

Table 2: Univariate logistic showing the effects of each prognostic indicator on treatment success

Covariates	6 weeks		12 weeks		26 weeks		52 weeks	
	OR (95 % CI)	P-value	OR (95 % CI)	P-value	OR (95 % CI)	P-value	OR (95 % CI)	P-value
Age	0.99 (0.95, 1.03)	0.57	1.00 (0.96, 1.04)	0.94	0.96 (0.92, 1.01)	0.11	1.00 (0.96, 1.04)	0.90
Female (ref: male)	1.20 (0.58, 2.50)	0.62	0.81 (0.40, 1.66)	0.57	0.95 (0.39, 2.31)	0.91	0.67 (0.30, 1.51)	0.34
Marital status (ref: unmarried/widow(er))								
Married/cohabiting	0.91 (0.40, 2.11)	0.83	0.47 (0.21, 1.08)	0.08	1.30 (0.49, 3.47)	0.60	1.36 (0.54, 3.44)	0.52
Level of education x time (ref. primary or secondary school)								
College or university	0.69 (0.31, 1.54)	0.36	0.48 (0.22, 1.05)	0.07	0.54 (0.19, 1.51)	0.24	0.97 (0.41, 2.29)	0.95
Excercises regularly	0.54 (0.26, 1.13)	0.10	0.58 (0.29, 1.17)	0.13	2.13 (0.86, 5.27)	0.10	1.22 (0.55, 2.74)	0.62
Paid work	0.74 (0.26, 2.06)	0.56	0.22 (0.07, 0.72)	0.01	1.08 (0.28, 4.18)	0.91	0.15 (0.02, 1.19)	0.07
Manual work	1.06 (0.49, 2.29)	0.88	1.16 (0.55, 2.45)	0.69	1.34 (0.53, 3.42)	0.54	0.18 (0.07, 0.50)	< 0.01
On paid sick-leave now	3.14 (1.37, 7.16)	0.01	2.10 (0.93, 4.75)	0.08	0.77 (0.28, 2.09)	0.61	0.55 (0.22, 1.32)	0.18
Duration of complaints (in weeks)	0.98 (0.88, 1.10)	0.77	0.97 (0.86, 1.09)	0.60	0.98 (0.85, 1.13)	0.77	1.13 (0.99, 1.31)	0.08
Dominant elbow affected	0.83 (0.38, 1.84)	0.65	1.58 (0.72, 3.49)	0.25	1.54 (0.58, 4.12)	0.39	2.19 (0.94, 5.11)	0.07
Pain every day last week	0.74 (0.13, 4.28)	0.74	1.11 (0.18, 6.96)	0.91	0.54 (0.04, 6.96)	0.64	-	
Use of analgetics last week	1.72 (0.76, 3.91)	0.20	1.58 (0.69, 3.63)	0.28	2.22 (0.80, 6.18)	0.13	0.86 (0.34, 2.14)	0.74
Acute start of symptoms	0.78 (0.38, 1.62)	0.51	0.99 (0.49, 2.00)	0.97	1.43 (0.58, 3.51)	0.44	0.97 (0.43, 2.17)	0.94
Similar complaints earlier	1.61 (0.69, 3.78)	0.27	0.76 (0.32, 1.78)	0.52	0.35 (0.12, 0.97)	0.04	0.69 (0.27, 1.76)	0.44
Probable overuse, usual activity	0.68 (0.32, 1.44)	0.31	0.61 (0.29, 1.28)	0.19	0.77 (0.31, 1.92)	0.58	0.20 (0.07, 0.59)	< 0.01
Probable overuse, unusual activity	1.55 (0.74, 3.26)	0.25	1.77 (0.85, 3.71)	0.13	1.44 (0.57, 3.59)	0.44	4.91 (1.68, 14.41)	< 0.01
Patients preference for treatment: Physiotherapy	1.68 (0.80, 3.54)	0.17	1.13 (0.55, 2.34)	0.74	0.64 (0.25, 1.63)	0.35	0.61 (0.27, 1.37)	0.23
Patients preference for treatment: Injection	1.24 (0.52, 2.96)	0.62	1.58 (0.67, 3.74)	0.30	0.71 (0.27, 1.87)	0.49	2.58 (0.79, 8.47)	0.12
Patients preference for treatment: Wait-and-see	0.12 (0.01, 1.12)	0.06	0.36 (0.07, 1.78)	0.21	2.77 (0.26, 29.83)	0.40	0.36 (0.08, 1.68)	0.19
Patients preference for treatment: No preference	0.75 (0.34, 1.65)	0.47	0.84 (0.40, 1.78)	0.66	1.98 (0.73, 5.35)	0.18	1.20 (0.50, 2.90)	0.68
Pain score on VAS	1.00 (0.99, 1.02)	0.62	0.99 (0.97, 1.01)	0.16	0.97 (0.94, 0.99)	0.01	0.97 (0.95, 0.99)	0.01
Affected function on VAS	0.93 (0.91, 0.96)	< 0.01	0.93 (0.91, 0.95)	< 0.01	0.90 (0.87, 0.94)	< 0.01	0.92 (0.89, 0.94)	< 0.01
Overall complaints on VAS	0.93 (0.90, 0.95)	< 0.01	0.91 (0.88, 0.93)	< 0.01	0.89 (0.85, 0.93)	< 0.01	0.89 (0.86, 0.92)	< 0.01
Pain free grip strength ratio	0.73 (0.18, 2.94)	0.66	0.43 (0.11, 1.72)	0.23	1.75 (0.31, 9.80)	0.53	0.55 (0.11, 2.72)	0.47
Maximum grip strength ratio	0.41 (0.12, 1.34)	0.14	0.54 (0.18, 1.60)	0.27	0.99 (0.20, 4.88)	0.99	1.04 (0.35, 3.13)	0.94
Pain Free Function Index	0.43 (0.31, 0.60)	< 0.01	0.51 (0.40, 0.66)	< 0.01	0.47 (0.34, 0.64)	< 0.01	0.57 (0.46, 0.72)	< 0.01
Pain free isometric (wrist): ref. None								
Some or distinct pain	0.04 (0.01, 0.14)	< 0.01	0.19 (0.06, 0.55)	< 0.01	0.13 (0.04, 0.44)	< 0.01	0.07 (0.02, 0.23)	< 0.01
Pain free isometric(finger): ref. None								
Some or distinct pain	0.15 (0.06, 0.34)	< 0.01	0.19 (0.08, 0.43)	< 0.01	0.35 (0.14, 0.87)	0.02	0.16 (0.07, 0.37)	< 0.01
