# Supplementary material for: Predictors for outcome in acute lateral epicondylitis
Source: BMC Musculoskelet Disord. 2019 Aug 17;20:375. doi: 10.1186/s12891-019-2758-y (PMC6698329; doi:10.1186/s12891-019-2758-y)
Supplement: Supplementary file 2 — Table S3. Adjusted multilevel logistic regression showing the effects of each prognostic indicator on treatment success (based on P ≤ 0.05 from the univariate analysis). (PDF 96 kb) [file 12891_2019_2758_MOESM2_ESM.pdf]

Table 3 Adjusted multilevel logistic regression showing the effects of each prognostic indicator on treatment success (based on P ≤ 0.05 from the univariate analysis)

	6 weeks		12 weeks		26 weeks		52 weeks	
**Covariates at baseline**	**OR (95 % CI)**	**P-value**	**OR (95 % CI)**	**P-value**	**OR (95 % CI)**	**P-value**	**OR (95 % CI)**	**P-value**
Age	0.94 (0.87, 1.02)	0.16	1.00 (0.94, 1.05)	0.92	1.00 (0.93, 1.07)	0.96	0.95 (0.89, 1.03)	0.20
Female (ref: Male)	2.50 (0.72, 8.69)	0.15	1.04 (0.37, 2.92)	0.94	3.23 (1.01, 10.28)	0.05	1.08 (0.37, 3.20)	0.89
On paid work	0.87 (0.03, 21.66)	0.93	0.53 (0.02, 11.42)	0.68	0.53 (0.02, 11.42)	0.68	0.53 (0.02, 11.41)	0.68
Manual labor	0.66 (0.18, 2.44)	0.53	2.01 (0.66, 6.12)	0.22	1.78 (0.51, 6.26)	0.37	0.33 (0.09, 1.13)	0.08
On paid sick leave at time of follow up	6.15 (1.42, 26.60)	0.02*	5.09 (1.45, 17.88)	0.01*	0.71 (0.18, 2.75)	0.62	0.84 (0.25, 2.80)	0.78
Similar complaints earlier	5.94 (1.52, 23.19)	0.01*	0.71 (0.22, 2.32)	0.57	0.20 (0.05, 0.89)	0.03*	0.68 (0.20, 2.36)	0.54
Probable overuse, unusual activity	2.82 (0.77, 10.25)	0.12	3.70 (1.11, 12.33)	0.03*	1.77 (0.50, 6.29)	0.38	1.78 (0.52, 6.03)	0.36
Pain score on VAS	1.01 (0.97, 1.05)	0.52	1.00 (0.97, 1.03)	0.89	0.99 (0.96, 1.02)	0.60	0.99 (0.96, 1.03)	0.71
Affected function on VAS	1.03 (0.99, 1.07)	0.07	1.01 (0.98, 1.03)	0.71	0.98 (0.94, 1.01)	0.18	1.02 (0.98, 1.05)	0.33
Overall complaints on VAS	1.01 (0.96, 1.05)	0.76	0.98 (0.95, 1.02)	0.31	1.02 (0.98, 1.06)	0.39	0.97 (0.93, 1.02)	0.24
Pain Free Function Index	0.42 (0.28, 0.63)	< 0.01*	0.58 (0.45, 0.75)	< 0.01*	0.52 (0.37, 0.72)	< 0.01*	0.75 (0.61, 0.94)	0.01*
Pain free isometric (wrist): ref. None								
Some or distinct pain	0.15 (0.02, 1.10)	0.06	0.49 (0.07, 3.19)	0.45	0.56 (0.13, 2.39)	0.44	0.22 (0.05, 1.01)	0.06
Pain free isometric(finger): ref. None								
Some or distinct pain	0.61 (0.14, 2.75)	0.52	0.67 (0.20, 2.29)	0.53	0.69 (0.16, 2.87)	0.61	0.50 (0.16, 1.58)	0.24

***) p<0.05**
