# Supplementary material for: Predictors for outcome in acute lateral epicondylitis
Source: BMC Musculoskelet Disord. 2019 Aug 17;20:375. doi: 10.1186/s12891-019-2758-y (PMC6698329; doi:10.1186/s12891-019-2758-y)
Supplement: Supplementary file 3 — Table S4. Univariate multilevel linear regression (MLR) showing the effects of each prognostic indicator on Pain Score (VAS) at each study time point after adjusting for baseline pain on VAS. (PDF 114 kb) [file 12891_2019_2758_MOESM3_ESM.pdf]

Table 4 Univariate multilevel linear regression (MLR) showing the effects of each prognostic indicator on Pain Score (VAS) at each study time point after adjusting for baseline pain on VAS

	6 weeks		12 weeks		26 weeks		52 weeks	
**Covariates**	**β (95 % CI)**	**P-value**	**β (95 % CI)**	**P-value**	**β (95 % CI)**	**P-value**	**β (95 % CI)**	**P-value**
Age	0.002 (-0.34, 0.35)	0.99	-0.06 (-0.40, 0.29)	0.74	0.45 (0.02, 0.87)	0.04	-0.08 (-0.39, 0.23)	0.61
Female (ref: male) x time	-1.86 (-8.74, 5.03)	0.60	0.02 (-6.87, 6.90)	1.00	3.74 (-4.76, 12.23)	0.39	0.05 (-6.17, 6.27)	0.99
Marital status (ref: unmarried or widow(er)) x time								
Married/cohabiting	-5.40 (-13.29, 2.49)	0.18	10.73 (2.93, 18.53)	0.01	-0.54 (-9.93, 8.85)	0.91	0.31 (-7.00, 7.61)	0.94
Level of education x time (ref. primary or secondary school)								
College or University	-5.19 (-12.59, 2.21)	0.17	0.83 (-6.55, 8.20)	0.83	-0.77 (-10.78, 9.24)	0.88	-4.61 (-11.15, 1.92)	0.17
Excercises regularly	4.54 (-2.21, 11.29)	0.19	-2.93 (-9.66, 3.79)	0.39	-5.61 (-14.03, 2.81)	0.19	-0.63 (-6.65, 5.40)	0.84
On paid work	6.51 (-3.33, 16.35)	0.19	14.99 (4.82, 25.17)	< 0.01	-8.02 (-20.89, 4.85)	0.22	4.27 (-4.51, 13.06)	0.34
Manual labour	3.24 (-3.78, 10.25)	0.37	0.05 (-7.02, 7.13)	0.99	-3.83 (-12.64, 4.98)	0.40	6.17 (-0.07, 12.40)	0.06
On paid sick-leave now	-3.76 (-11.40, 3.88)	0.33	-3.24 (-10.88, 4.40)	0.41	1.49 (-7.80, 10.79)	0.75	0.09 (-6.96, 7.15)	0.98
Duration of complaints in weeks	0.27 (-0.82, 1.35)	0.63	-0.19 (-1.30, 0.92)	0.74	1.35 (0.03, 2.66)	0.04	-0.10 (-1.12, 0.92)	0.85
Dominant elbow affected	-0.42 (-7.87, 7.03)	0.91	2.84 (-4.72, 10.40)	0.46	-1.91 (-11.26, 7.44)	0.69	-1.08 (-7.78, 5.61)	0.75
Pain every day last week	12.14 (-5.01, 29.28)	0.17	8.72 (-8.25, 25.68)	0.31	-1.03 (-23.45, 21.39)	0.93	2.42 (-12.62, 17.46)	0.75
Use of analgesics last week	-0.32 (-7.86, 7.22)	0.93	3.72 (-3.96, 11.40)	0.34	3.97 (-5.26, 13.21)	0.40	3.12 (-3.89, 10.13)	0.38
Acute start of symptoms	6.15 (-0.61, 12.91)	0.08	3.26 (-3.54, 10.06)	0.35	-9.40 (-17.92, -0.87)	0.03	4.95 (-1.12, 11.02)	0.11
Similar complaints earlier	-5.86 (-13.96, 2.25)	0.16	-1.09 (-9.29, 7.11)	0.80	10.49 (0.87, 20.11)	0.03	2.02 (-5.40, 9.45)	0.59
Probable over-use usual activity	4.60 (-2.34, 11.55)	0.19	2.91 (-4.11, 9.93)	0.42	6.17 (-2.39, 14.73)	0.16	8.79 (2.45, 15.14)	0.01
Probable over-use unusual activity	-5.46 (-12.40, 1.48)	0.12	-4.19 (-11.22, 2.83)	0.24	-7.00 (-15.59, 1.58)	0.11	-8.71 (-15.03, -2.38)	0.01
Patients preference for treatment: Physiotherapy	-5.96 (-12.91, 0.98)	0.09	-2.80 (-9.77, 4.17)	0.43	8.48 (-0.39, 17.36)	0.06	0.26 (-5.92, 6.44)	0.94
Patients preference for treatment: Injection	2.96 (-5.27, 11.19)	0.48	-2.15 (-10.37, 6.07)	0.61	2.45 (-6.92, 11.81)	0.61	-4.42 (-12.06, 3.22)	0.26
Patients preference for treatment: Wait and see	-0.61 (-14.43, 13.21)	0.93	4.26 (-10.74, 19.26)	0.58	-8.87 (-27.30, 9.57)	0.35	10.27 (-2.83, 23.37)	0.12
Patients preference for treatment: No preference	4.06 (-3.17, 11.29)	0.27	2.89 (-4.29, 10.07)	0.43	-9.53 (-18.61, 0.45)	0.04	0.28 (-6.20, 6.75)	0.93
Baseline pain on VAS	0.45 (0.28, 0.61)	< 0.01	0.40 (0.24, 0.57)	< 0.01	0.46 (0.26, 0.67)	< 0.01	0.21 (0.05, 0.36)	0.01
Affected function on VAS	0.60 (0.51, 0.70)	< 0.01	0.67 (0.58, 0.77)	< 0.01	0.78 (0.65, 0.91)	< 0.01	0.73 (0.61, 0.84)	< 0.01
Overall complaints on VAS	0.73 (0.67, 0.79)	< 0.01	0.82 (0.76, 0.88)	< 0.01	0.95 (0.87, 1.04)	< 0.01	0.83 (0.76, 0.91)	< 0.01
Pain free grip strength ratio	-42.74 (-52.01, -33.48)	< 0.01	-29.52 (-37.90, -21.15)	< 0.01	-25.12 (-34.07, -16.17)	< 0.01	-31.94 (-41.46, -22.42)	< 0.01
Maximum grip strength ratio	-44.16 (-56.26, -32.06)	< 0.01	-28.42 (-40.24, -16.59)	< 0.01	-44.29 (-61.09, -27.49)	< 0.01	-28.05 (-43.88, -12.22)	< 0.01
Pain Free Function Index	6.49 (5.18, 7.81)	< 0.01	6.12 (4.93, 7.31)	< 0.01	6.62 (5.24, 8.00)	< 0.01	3.14 (2.27, 4.02)	< 0.01
Pressure-pain threshold	-20.59 (-32.28, -8.89)	< 0.01	-13.88 (-24.01, -3.74)	0,01	-30.73 (-42.66, -18.81)	< 0.01	-8.43 (-17.58, 0.72)	0.07
Pain free isometric (wrist): ref. None								
Some or distinct pain	22.44 (13.79, 31.09)	< 0.01	19.34 (10.97, 27.70)	< 0.01	20.15 (11.57, 28.74)	< 0.01	15.98 (10.21, 21.75)	< 0.01
Pain free isometric(finger): ref. None								
Some or distinct pain	20.62 (13.53, 27.70)	< 0.01	22.54 (15.59, 29.49)	< 0.01	16.39 (8.61, 24.17)	< 0.01	15.67 (9.54, 21.79)	< 0.01
