# Supplementary material for: Predictors for outcome in acute lateral epicondylitis
Source: BMC Musculoskelet Disord. 2019 Aug 17;20:375. doi: 10.1186/s12891-019-2758-y (PMC6698329; doi:10.1186/s12891-019-2758-y)
Supplement: Supplementary file 4 — Table S5. Adjusted MLR showing the effects of each prognostic indicator on pain (VAS) at each study time point (based on P ≤ 0.20 from the univariate analysis). (PDF 192 kb) [file 12891_2019_2758_MOESM4_ESM.pdf]

Table 5 Adjusted MLR showing the effects of each prognostic indicator on pain (VAS) at each study time point (based on P ≤ 0.20 from the univariate analysis)

	6 weeks		12 weeks		26 weeks		52 weeks	
**Covariates at baseline**	**β (95 % CI)**	**P-value**	**β (95 % CI)**	**P-value**	**β (95 % CI)**	**P-value**	**β (95 % CI)**	**P-value**
Age	0.14 (-0.10, 0.38)	0.25	0.08 (-0.15, 0.31)	0.48	0.14 (-0.10, 0.38)	0.26	-0.06 (-0.25, 0.14)	0.58
Female gender (ref. Male gender)	-1.55 (-5.89, 2.78)	0.48	-1.64 (-5.83, 2.54)	0.44	2.58 (-2.19, 7.35)	0.29	-3.81 (-7.25, -0.36)	0.03*
Marital status (ref: unmarried/ widow(er))								
Married/cohabiting	-4.43 (-9.01, 0.15)	0.06	1.84 (-3.29, 6.96)	0.48	1.34 (-4.13, 6.80)	0.63	1.40 (-2.59, 5.38)	0.49
Level of education x time (ref. primary or secondary school)								
College or University	-5.72 (-9.93, -1.50)	0.01*	-4.11 (-8.67, 0.44)	0.08	-2.53 (-8.48, 3.41)	0.40	-2.57 (-6.34, 1.21)	0.18
Exercises regularly	-0.68 (-4.55, 3.19)	0.73	1.48 (-2.33, 5.29)	0.45	-6.23 (-10.94, -1.53)	0.01*	-1.57 (-4.72, 1.57)	0.33
On paid work	12.78 (3.12, 22.43)	0.01*	5.81 (-6.15, 17.78)	0.34	-11.58 (-20.85, -2.31)	0.01*	0.26 (-6.27, 6.79)	0.94
Manual labor	-2.10 (-6.23, 2.02)	0.32	0.28 (-4.25, 4.81)	0.90	-3.13 (-8.36, 2.10)	0.24	-3.04 (-6.68, 0.60)	0.10
Duration of complaints in weeks	0.18 (-0.51, 0.87)	0.61	-0.25 (-0.93, 0.43)	0.47	0.32 (-0.44, 1.07)	0.41	0.05 (-0.53, 0.63)	0.87
Pain every day last week	1.52 (-9.67, 12.72)	0.79	0.23 (-9.40, 9.86)	0.96	1.90 (-9.69, 13.49)	0.75	-2.12 (-9.74, 5.50)	0.59
Acute start of symptoms	-1.30 (-5.47, 2.87)	0.54	2.01 (-2.38, 6.40)	0.37	-0.39 (-5.38, 4.59)	0.88	4.32 (0.86, 7.79)	0.01*
Similar complaints earlier	0.54 (-4.26, 5.33)	0.83	1.49 (-3.23, 6.21)	0.54	2.26 (-3.27, 7.79)	0.42	3.44 (-0.40, 7.28)	0.08
Probable over-use unusual activity	-2.69 (-7.11, 1.73)	0.23	2.87 (-1.63, 7.36)	0.21	-4.81 (-10.04, 0.42)	0.07	-3.53 (-7.11, 0.05)	0.06
Patients preference for treatment: Physiotherapy	1.06 (-4.02, 6.15)	0.68	2.93 (-2.64, 8.49)	0.30	-0.54 (-6.27, 5.18)	0.85	0.85 (-3.36, 5.07)	0.69
Patients preference for treatment: Wait and see	1.07 (-7.35, 9.50)	0.80	7.80 (-1.17, 16.77)	0.09	-7.16 (-18.23, 3.91)	0.21	2.31 (-5.53, 10.16)	0.56
Patients preference for treatment: No preference	-4.22 (-9.46, 1.02)	0.11	2.19 (-3.31, 7.70)	0.44	-0.71 (-6.38, 4.96)	0.81	1.04 (-3.40, 5.49)	0.65
Baseline pain VAS	0.23 (0.12, 0.33)	< 0.01*	0.05 (-0.06, 0.15)	0.39	0.04 (-0.07, 0.15)	0.44	0.06 (-0.02, 0.15)	0.13
Affected function on VAS	0.10 (-0.02, 0.22)	0.09	-0.02 (-0.16, 0.13)	0.81	-0.21 (-0.38, -0.04)	0.02*	0.08 (-0.08, 0.25)	0.32
Overall complaints on VAS	0.56 (0.44, 0.69)	< 0.01*	0.82 (0.67, 0.98)	< 0.01	1.04 (0.86, 1.22)	< 0.01*	0.59 (0.42, 0.77)	< 0.01*
Pain free grip strength ratio	-3.04 (-13.15, 7.08)	0.56	-1.40 (-8.73, 5.92)	0.71	-1.54 (-8.03, 4.94)	0.64	-5.50 (-14.06, 3.07)	0.21
Maximum grip strength ratio	-3.68 (-14.50, 7.14)	0.51	2.26 (-7.10, 11.63)	0.64	3.07 (-8.98, 15.12)	0.62	-2.98 (-12.34, 6.39)	0.53
Pain Free Function Index	0.89 (-0.54, 2.32)	0.22	0.57 (-0.72, 1.86)	0.39	0.57 (-0.87, 2.02)	0.44	0.53 (-0.24, 1.30)	0.18
Pain free isometric (wrist): ref. None								
Some or distinct pain	-1.56 (-8.77, 5.66)	0.67	-1.87 (-8.43, 4.69)	0.58	1.74 (-4.71, 8.19)	0.60	3.50 (-0.45, 7.44)	0.08
Pain free isometric (3. finger): ref. None								
Some or distinct pain	0.87 (-4.73, 6.47)	0.76	2.87 (-2.63, 8.38)	0.31	2.73 (-3.64, 9.10)	0.40	2.50 (-1.62, 6.61)	0.23

***) p<0.05**
